# Supplementary material for: HIV-1 interactions with sialic acid-binding bacterial lectins promote virus infectivity in vitro and mucosal transmission in humanized mice
Source: bioRxiv. 2026 May 6:2026.05.05.722898. Preprint. [Version 1] doi: 10.64898/2026.05.05.722898 (PMC13174408; doi:10.64898/2026.05.05.722898)
Supplement: 1 [file NIHPP2026.05.05.722898V1-supplement-1.pdf]

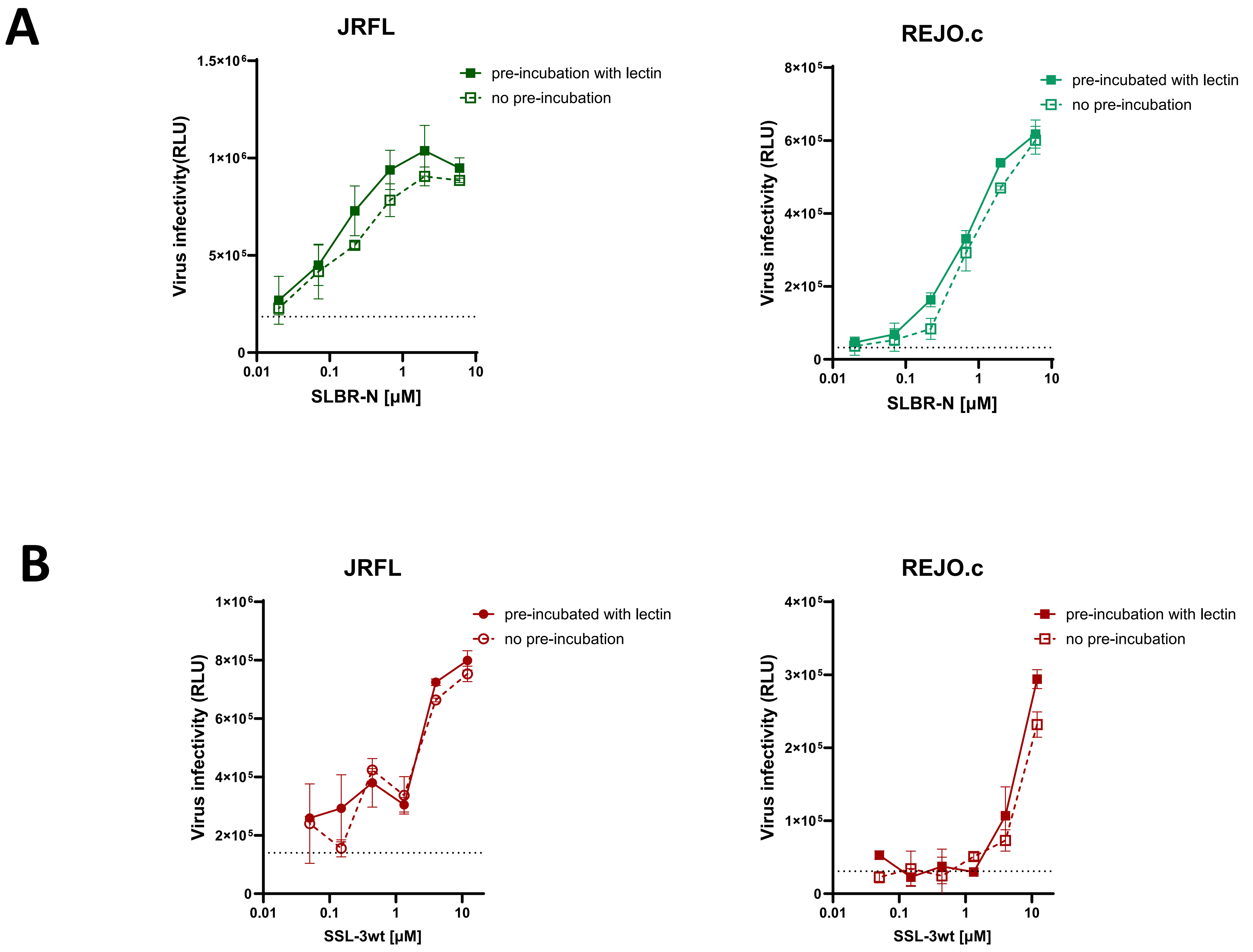

Supplemental Figure S1. SLBR-N and SSL3 lectins enhanced HIV-1 infectivity with or without lectin-virus preincubation.

JRFL and REJO.c IMC virions were either pre-incubated with SLBR-N (**A**) or SSL3 (**B**) for 1 hour at 37°C or added simultaneously to lectin and TZM-bl target cells without pre-incubation. Virus infectivity in TZM-bl reporter cells was quantified 48 hours post-infection. Mean ± SD are shown. RLU: relative luminescence unit. Dotted lines: virus infection without lectin.

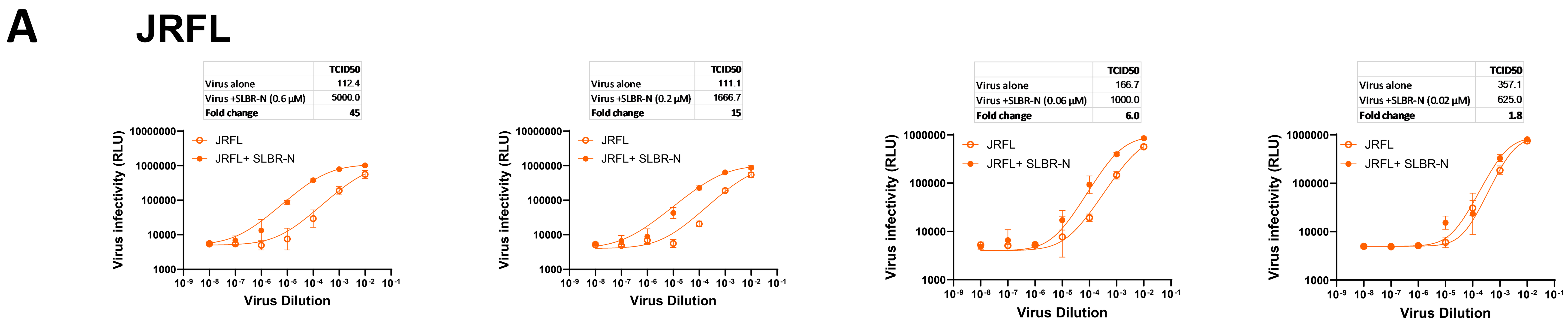

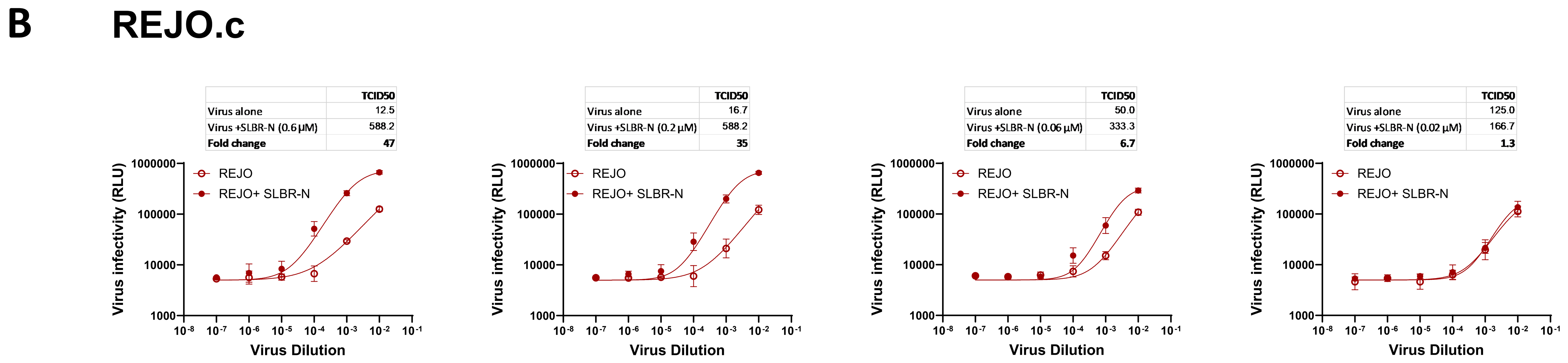

Supplemental Figure S2. SLBR-N increases virus infectivity as measured by TCID50.
Virions of JRFL (**A**) and REJO.c (**B**) IMC were serially diluted (10^{-2} to 10^{-7} or 10^{-8}), treated with SLBR-N (0.6, 0.2, 0.06, and 0.02 µM) or left untreated at 37°C for 1 hour and then incubated with TZM-bl reporter cells for 48 hours. Virus infection was measured by beta-galactosidase activity. Reciprocal virus dilutions to attain 50% infection (TCID50) were calculated in GraphPad Prism using a non-linear fit model. RLU: relative luminescence unit.
